# Arseniate of Quinia

**Published:** 1847-04

**Authors:** 


9. "*Arseniate of Quinia.*—This salt, first prepared by M. Bourières, has latterly been much used in France in the treatment of obstinate intermittents, and it is stated with much success; the chief obstacle to its more general employment being, according to Dr. Boudin, its extreme bitterness. It is readily prepared as follows: Dissolve half an ounce of sulphate of quinia in boiling water, and precipitate with ammonia; wash and dry the precipitate, and dissolve it with the aid of heat in three ounces of distilled water, containing two scruples of arsenious acid in solution; as the solution cools crystals of arseniate of quinia are deposited, which are to be dissolved in distilled water and recrystalized. It is a light, white salt, crystalized in brilliant, satiny needles. It is soluble in water, but more so in boiling than in cold water; it is also soluble in weak alcohol or in ether. The dose of it is from one to two grains in divided doses in the course of twenty-four hours. It is usually given in solution in distilled water, to which a little simple syrup may be added."—*Med. Exam.*, Oct., 1846.

